# Associations between Indicators of Livestock Farming Intensity and Incidence of Human Shiga Toxin-Producing *Escherichia Coli* Infection

**DOI:** 10.3201/eid0803.010159

**Published:** 2002-03

**Authors:** James E. Valcour, Pascal Michel, Scott A. McEwen, Jeffrey B. Wilson

**Affiliations:** University of Guelph, Guelph, Ontario, Canada; † Université de Montréal, St-Hyacinthe, Québec, Canada; ‡ University of Guelph, Guelph, Ontario, and Centre for Infectious Disease Prevention and Control, Health Canada, Ottawa, Ontario, Canada

**Keywords:** *Escherichia coli* 0157, STEC, livestock, spatial epidemiology, manure

## Abstract

The impact of livestock farming on the incidence of human Shiga toxin-producing *Escherichia coli* (STEC) infection was assessed by using several livestock density indicators (LDI) that were generated in a systematic approach. A total of 80 LDI were considered suitable proxy measures for livestock density. Multivariate Poisson regression identified several LDI as having a significant spatial association with the incidence of human STEC infection. The strongest associations with human STEC infection were the ratio of beef cattle number to human population and the application of manure to the surface of agricultural land by a solid spreader and by a liquid spreader showed. This study demonstrates the value of using a systematic approach in identifying LDI and other spatial predictors of disease.

Infection with Shiga toxin-producing *Escherichia coli* (STEC) is associated with a spectrum of illnesses including watery diarrhea, bloody diarrhea, and the hemolytic uremic syndrome, a potentially fatal condition characterized by acute renal failure (*1*). Although a variety of E. coli serotypes have been associated with human illness, the most important among these is O157:H7. Cattle are the principal reservoir for these organisms. Important sources of infection include consumption of undercooked hamburger and other contaminated food products and direct or indirect contact with infected persons (*2*–*4*).

Recent studies suggest that direct or indirect exposure to cattle are important potential sources of infection (*2*,*3*). Among this evidence is the finding of Michel et al. that the incidence of human STEC infection was higher in rural areas than in urban areas of Ontario, Canada. By using spatial regression analysis, these investigators demonstrated a strong association between the incidence of human STEC infection and cattle density, expressed as total number of cattle per hectare. Potential routes of infection in rural settings include direct contact with cattle (*2*,*5*), consumption of raw milk (*2*,*4*), contamination of well water with agricultural runoff (*6*,*7*), and contamination of locally produced food products (*8*).

The approach taken by Michel et al. (*3*) demonstrated the value of spatial analysis for identifying areas at high risk for STEC infection and for elucidating potential risk factors. The objective of the present study was to develop a systematic approach to creating and evaluating spatial measures of livestock density (livestock density indicators, or LDI) as a means of identifying those best suited to assessing the impact of livestock farming activities on the incidence of human STEC infection.

## Methods

### Data

Data on 1,276 cases of human STEC infection reported in Ontario from January 1996 to December 1998 were obtained from the Reportable Disease Information System (RDIS) of the Ontario Ministry of Health and Long Term Care. Infection with STEC is notifiable in Ontario, and more than 95% of reported cases are due to *E. coli* O157:H7 (*3*). Cases were excluded if they were identified as part of a communitywide outbreak resulting from a single source, such as contaminated water supply.

Consolidated census subdivision (CCS) identifiers were added to the database via a software package that links CCS to appropriate postal codes (Postal Code Conversion File; Statistics Canada). The human population distribution of Ontario was obtained from GeoRef 1996 Census (Statistics Canada), which is based on data collected from all households in the province. CCS areas, which are coded in square kilometers, were also extracted from the GeoRef database. Livestock distribution and land use data were collected from the 1996 Census of Agriculture (Statistics Canada), and area units were converted from hectares to square kilometers. Information on soil development and drainage characteristics for Ontario were obtained from the Canadian Soil Information System (CANSIS) website (http://res.agr.ca/cansis/). All data were aggregated to the CCS because this was the most detailed level at which agricultural data were available.

The study area incorporated 435 townships in the southern area of the province. CCS data from the northern portion of the province were excluded to avoid a potential bias, since this area was sparsely populated and had little agricultural activity. No information was available on confidential records from the 1996 Census of Agriculture.

### STEC Incidence Rates

Incidence rates were determined over a 3-year period, from 1996 to 1998, and were expressed as the number of STEC cases per 100,000 population per year in Ontario CCS.

### Spatial Analysis

ArcView version 3.1 (ESRI, Redlands, CA, USA) was used to create chloropleth maps based on disease rates and LDI measures. For the purposes of mapping, the method of nested means, as adapted by Michel and colleagues (*9*), was used to classify incidence rates. Quintile breaks in the data were used to catergorize continuous LDI measures (for example, the lower 20% quintile for the ratio of beef cattle to human population is 0.000 to 0.049). ArcView was also used to calculate CCS centroid locations (latitude and longitude) to allow calculation of autocorrelation measures. SpaceStat version 1.9 (Regional Research Institute, West Virginia University, Morgantown, WV, USA) was used to calculate the Euclidean distance between CCS centroids, so that an inverse square distance matrix could be produced. This matrix was used in the calculation of Moran’s *I* and *G_i_* statistics for the 435 townships in the study area.

Soil landscape coverage version 2.2 and hydrological data version 2.2 were downloaded and imported into ArcView. Attributes containing information on the dominant soil type and drainage characteristics of the soil were mapped. ArcView’s GeoProcessing extension was used to clip overlay analysis that would remove areas normally covered with bodies of water.

The Spatial Analyst extension of ArcView was used to perform a cross-tab query to obtain soil type and drainage characteristic in each CCS. Soil typing within each CCS was based on the predominent type of soil; if two or more soil or drainage types occupied equal areas within a township, then the variable was set to a null value. Soil development and drainage characteristics were based on the Canadian system of soil classification (*10*).

### Development of Livestock Density Indicators

For this study, an LDI was defined as a measure of livestock farming intensity that captures information on the number of animals or the amount of their fecal waste relative to various agricultural and environmental factors within a given geographic area. LDIs were created by combining a series of variables that were considered a priori to be potentially spatially associated with human STEC infection, based on a comprehensive analysis of possible sources and pathways of infection. Variables were constrained by their availability in existing databases.

We grouped these variables into “dimensions” and “components” (Table 1). Dimensions included variables related to number of animals, area of manure application, land uses, and human population within a given CCS. Within the dimensions were components that provided further refinement. For example, components within the dimension “animal” included numbers of various animal species within a given CCS, while components within “manure” consisted of specific manure characteristics and methods of application (Table 1).

**Table 1:** Dimensions and components used in creating livestock density indicators to predict incidence of human shiga toxin-producing *Escherichia coli* infection, Ontario

Dimension	Component
Animal	Total no. of cattle in CCS^a^
	Total no. of dairy cattle in CCS
	Total no. of beef cattle in CCS
	Total no. of chickens in CCS
	Total no. of pigs in CCS
	
Manure	Area of CCS having manure applied via solid spreader
	Area of CCS having manure applied via irrigation system
	Area of CCS having manure applied to soil surface via liquid spreader
	Area of CCS having manure injected into soil via liquid spreader
	
Land use	Total CCS area
	Absolute or % area of farm land in CCS
	Absolute or % area of pasture land in CCS
	Absolute or % area of crop land in CCS
	
Human population	Total human population in CCS

^a^CCS = consolidated census subdivision. Area of CCS is in square kilometers; absolute = absolute squar kilometers.

Dimensions were combined mathematically according to equations denoted as “frames” (Table 2) to form the LDI. Within each frame, all possible combinations of the relevant components were used. For example, Frame 3 (human population/manure) was used to create four separate LDI consisting of the ratios of total human population in a CCS to the area having manure applied either 1) by solid spreader, 2) by irrigation, 3) as liquid on the soil surface, or 4) as liquid by injection into the soil. Each generated LDI was examined for biological and logical plausibility, and those considered inappropriate were discarded.

**Table 2:** Frames used developing livestock density indicators for predicting incidence of human shiga toxin-producing *Escherichia coli* infection, Ontario

Frame no.	Equation	Example
1	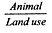	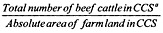
		
2	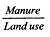	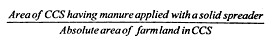
		
3	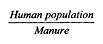	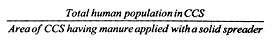
		
4	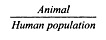	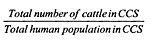
		
5	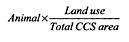	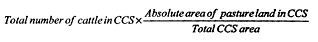
		
6	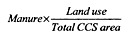	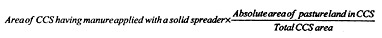

^a^CCS = consolidated census subdivision.

### Statistical Analysis

Data manipulation, merging of data sets, and statistical analyses were conducted by using the Statistical Analysis System for personal computers, version 6.12 (SAS Institute Inc., Cary, NC, USA). Univariate associations between each indicator and the incidence of human STEC infection were examined by using Poisson regression analysis. The GLIMMIX macro in SAS was used, and census division was entered as a repeated effect to induce a correlation structure in an attempt to control for the spatial effects inherent in the data.

Variable selection was performed in several steps. Initially, LDI were grouped along common components in their numerator (e.g., all LDI with dairy cattle in the numerator were combined into a single group). LDI within each group were then entered into a separate multivariate model, and non-significant LDI were removed by backward elimination until a minimum of one variable remained (stage 1 models). This procedure reduced the potential number of variables offered to subsequent models.

Variables thus identified were then offered to a second series of multivariate models (stage 2 models), each of which was subjected to a backward elimination procedure. Variables offered to stage 2 models consisted of all combinations of one variable from each of the models developed in stage 1.

Moran’s *I* and *G_i_* statistics were calculated for STEC incidence rates, which provided a measure of overall and local spatial autocorrelation. For all statistical analyses, a significance level of 5% was used (p=0.05).

## Results

**Figure 1:**
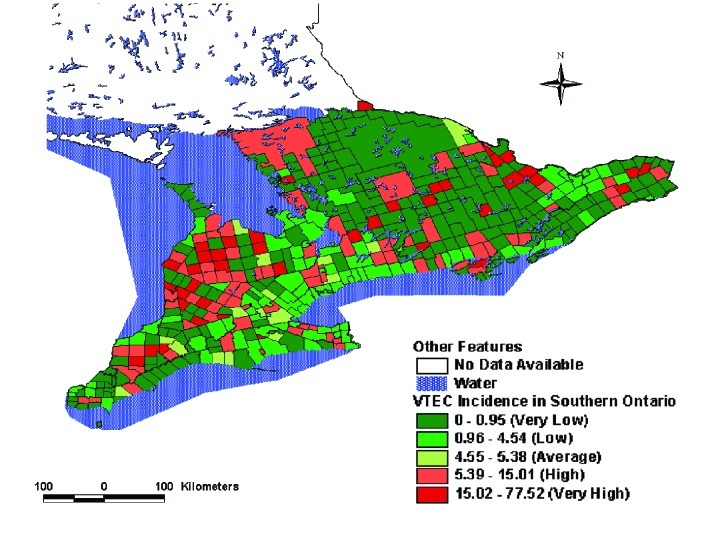
Yearly incidence of shiga toxin-producing *Escherichia coli* infection (per 100,000 population), southern Ontario, 1996-1998.

Geographic distribution of the yearly incidence of human STEC infection in Ontario between 1996 and 1998 is shown in Figure 1. According to the nested means techniques, STEC incidence rates were classified as very low incidence (0.00 to 0.95 per 100,000) in 204 CCS areas, low (0.96 to 4.54 per 100,000) in 95, average (4.55 to 5.38 per 100,000) in 15, high (5.39 to 15.01 per 100,000) in 78, and very high (15.02 to 77.52 per 100,000) in 41. CCS where the incidence of STEC infection was classified as high or very high were located primarily in the northwestern portion of southern Ontario, with smaller numbers in eastern Ontario.

Moran’s *I* calculation for the incidence of STEC infection indicated a significantly positive autocorrelation (p=0.012). *G_i_* statistics for the CCS areas with the 10 highest and 10 lowest incidence rates were also statistically significant (p<0.003).

A total of 8,316 LDI were generated, of which all but 80 were eliminated on the basis of biological plausibility. Of these 80 variables, 33 had a significant univariate association with the incidence of human STEC infection. Of these 33, 9 (27.3%) were based on the number of beef cattle, the total number of cattle per CCS, and measures of manure application; 4 (12.1%) were based on the number of standardized animal units per CCS; and 1 (3.0%) was based on both the number of dairy cattle and chickens per CCS.

The number of sheep or goats, soil type, or drainage characteristics were not significantly associated with the incidence of human STEC infection in the univariate analysis. The 10 LDI having the highest r^2^ values in univariate analyses are shown in Table 3. All of these LDI were based on either the number of cattle, beef cattle, or animal units per CCS.

**Table 3:** Top competing individual livestock density indicators for predicting the incidence of human shiga toxin-producing *Escherichia coli* infection, Ontario, 1996–1998

Livestock density indicator ^a^	Estimate	S.E.	r^2^
Ratio of beef cattle to human population Intercept	0.6872 1.4634	0.1384 0.1018	0.099
No. of beef cattle per km^2^ township area Intercept	0.0998 1.1645	0.0230 0.1674	0.098
No. of beef cattle per km^2^ weighted farmland (farmland/township area) Intercept	0.0004 1.3085	0.0001 0.1430	0.076
No. of beef cattle per km^2^ weighted pasture land (pasture land/township area) Intercept	0.0014 1.4449	0.0003 0.1182	0.060
Total no. of cattle per km^2^ of township area Intercept	0.0154 1.2217	0.0034 0.1355	0.053
Total no. of cattle per km^2^ weighted pasture land (pasture land/township area) Intercept	0.0003 1.4165	0.0001 0.1003	0.046
Total no. of cattle per km^2^ farmland Intercept	0.0139 1.0532	0.0036 0.1834	0.045
Animal units per km^2^ township area Intercept	0.0129 1.2548	0.0030 0.1317	0.044
Ratio of total number of cattle to human population Intercept	0.1477 1.3878	0.0344 0.1050	0.043
Number of beef cattle Intercept	0.0003 1.3515	0.0001 0.1540	0.041

^a^All LDI significant at p>0.001.

Multivariate modeling resulted in the creation of 16 unique stage 2 models with r^2^ values ranging from 0.0932 to 0.266. The multivariate model having the highest r^2^ value, shown in Table 4, consisted of HUMBCOW (the ratio of the number of beef cattle to the human population in a CCS), PIGFARM (the total number of swine per km^2^ of farm land in a CCS), SMANCCS (the proportion of land in a CCS in which manure is applied by a solid spreader), and LSMANCRP (the proportion of cropland in a CCS in which liquid manure is applied to the soil surface. HUMBCOW, SMANCCS, and LSMANCRP were all positively (and independently) associated with the incidence of human STEC infection, whereas PIGFARM was negatively associated.

**Table 4:** Multivariable spatial Poission regression models for predicting incidence of human shiga-toxin-producing *Escherichia coli* infection, Ontario, 1996-1998

Variable	Estimate	S.E.	p-value	r^2^
Intercept HUMBCOW^a^ PIGFARM^b^ SMANCCS^c^ LSMANCRP^d^	1.04 0.65 -0.003 4.19 7.82	0.18 0.13 0.001 1.86 2.47	<0.001 <0.001 0.04 0.03 0.002	0.2655

^a^Ratio of no. beef cattle to human population in a consolidated census subdivision (CCS). ^b^Total no. of swine per km^2^ of farmland in a CCS. ^c^Proportion in a CCS having manure applied to land surface via solid spreader. ^d^Proportion in a CCS having manure applied to land surface via liquid spreader.

## Discussion

The results of our analyses are consistent with the findings of Michel et al (*3*), who demonstrated a higher incidence of human STEC infection in rural areas of Ontario, as opposed to urban areas, and a spatial association between the incidence of human STEC infection and cattle density. These findings are also consistent with other reports in the literature, including outbreaks of STEC infection related to consumption of unpasteurized milk (*2*) and water from shallow wells, direct contact with cattle (*5*), and an association between endemic STEC infection and exposure to agricultural environments (*2*,*11*).

To our knowledge, this is the first time the application of manure to land has been identified as a potential risk factor for endemic human STEC infection. Runoff from agricultural land that has been treated with manure has the potential to contaminate local surface water and wells that supply water for human consumption (*12*).

A relationship between agricultural activities, such as manure spreading, animal density, and elevated fecal bacterial counts in local streams. was demonstrated in 1989 by Meals (*13*). An outbreak of STEC infection in New York state was associated with contaminated well water used in the preparation of beverages and ice at a county fair (*6*). It was thought that the well in question became contaminated with manure-laden water as a result of recent heavy rains.

More recently, contamination of a municipal water supply with *E. coli* O157:H7 and *Campylobacter* spp. in Walkerton, Ontario, Canada, resulted in the largest documented outbreak of gastroenteritis caused by multiple pathogens. Strong evidence suggests that contamination of Walkerton’s water supply was due to manure runoff from a nearby farm that entered a shallow well supplying the municipal water system (*14*).

The density of swine within a CCS was negatively associated with the incidence of human STEC infection. This apparent protective effect may simply be the result of a relative absence of cattle in areas where swine are intensively farmed. Although swine commonly harbor STEC within their intestinal tract, they are not considered to be important reservoirs of E. coli O157:H7 (*15*). Past studies have identified sheep and goats as important reservoirs for STEC (*15*,*16*), but these animals were not identified as important predictors of human STEC infection in our study. One explanation may be the relatively low numbers of these animals compared with other livestock types.

This study demonstrates the value of using a systematic approach to identifying potential LDI. The approach enabled us to examine a large pool of potential covariates from which appropriate indicators could be assessed and used to evaluate the association between livestock intensity and incidence of human STEC infection. The chosen indicators were biologically plausible and allowed for identification of a previously unreported risk factor.

**Figure 2:**
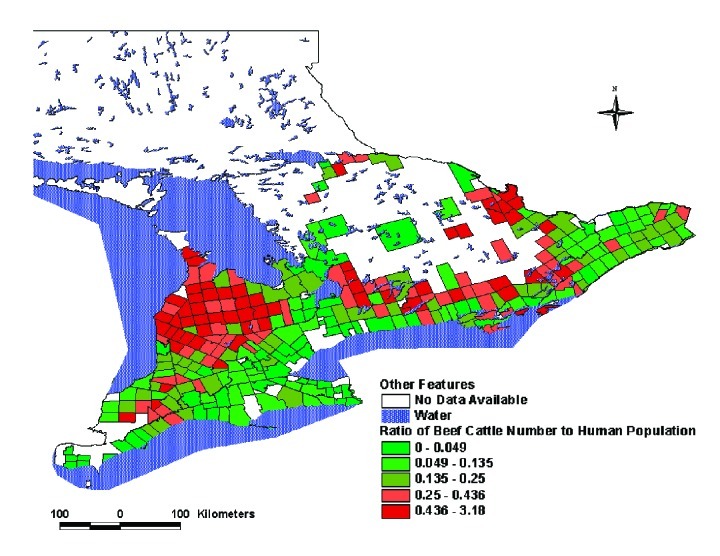
Ratio of beef cattle to human population (number of animals per person), southern Ontario, 1996).

**Figure 3:**
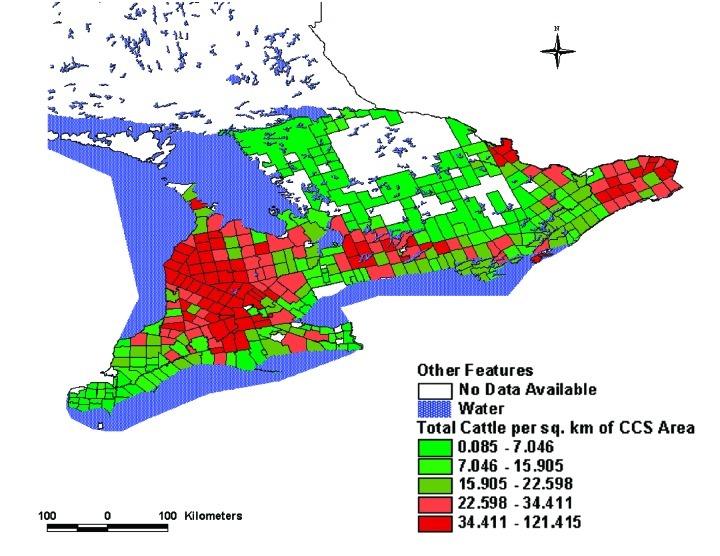
Total number of cattle per square kilometers, southern Ontario, 1996.

By using a systematic construction, we identified LDI that were more strongly associated with the incidence of STEC infection than has been reported previously (*3*). When modeled at the same geographic scale, the r^2^ value for the best model from our investigation (i.e., the ratio of beef cattle to human population as a measure of cattle density) was 0.27 compared with 0.14 for the total cattle density model used in Michel’s report (*3*). These differences in r^2^ values may be the result of our selecting beef cattle for the LDI, rather than total number of cattle. It is worth noting that this difference in association is not necessarily evident from maps (compare Figures 2 and 3), because both indicators suggest roughly similar distributions of cattle density, with the greatest concentration in CCS located in south-central and eastern Ontario.

Caution should be exercised when interpreting our study results, however, because not all potential confounding variables (e.g., age or gender of the infected humans) were included in the analysis. Also, systematic errors arising from differential reporting rates may have biased the relationship between the incidence of human STEC infection and the risk factors studied. Since several LDI were investigated, some associations we observed may have arisen from chance alone.

Through linkage of existing data sources, spatial analytic techniques provide a means of identifying populations at high risk and potential risk factors for STEC infection. The approach outlined in this study provides a rational, practical, and powerful tool for public health. As spatial analysis becomes more widely used in epidemiology, we anticipate that the development of such approaches will take on increasing importance.
